# Surveillance of violence through health service interactions in Wales: A longitudinal national data linkage study.

**DOI:** 10.23889/ijpds.v9i5.2606

**Published:** 2024-09-18

**Authors:** Jane Lyons, Harri Doel, Richard Fry, Rhodri Johnson, Arron Lacey, Huw Strafford, Samantha Turner, Ronan Lyons, Lucy Griffiths

**Affiliations:** 1 Swansea University

## Objective and Approach

The study aimed to utilise multiple population-wide linked administrative, health, and survey data sources to identify victims from violence across various sociodemographic groups using healthcare service interactions. We calculated violence rates for the population of Wales to identify groups of people that experience higher rates of violence compared to the general population.

Our approach involved creating an observational population-wide e-cohort utilising data held in the Secure Anonymised Information Linkage (SAIL) Databank. General practice records, emergency department attendances, hospital admissions, and the Office of National Statistics (ONS) deaths register were linked to the Welsh Demographic Service dataset to include individuals living in Wales, UK between 2013-2022, allowing for a 10-year analysis.

## Results

In total, 3.3 million individuals were included in the study, with 80,079 individuals having interacted with health services or died due to violence over the 10-year period. On average, every 4 per 1000 of the study population had experienced violence (2013-2022) with the majority of these being captured through emergency department attendances. 55% were male, of which 71% were aged under 35. The majority of individuals lived in the more deprived areas of Wales (61%).

## Conclusions and Implications

It is important to understand and quantify violence over time to produce insight into which sociodemographic groups of individuals are being disproportionately impacted by violence for designing disparity-reducing policy interventions. ONS Census 2021 data will be used to examine the impact of violence by protected characteristics (e.g. disability, race, sexual orientation) under the UK Equality Act 2010.

